# Association of coffee consumption with risk of colorectal cancer: a meta-analysis of prospective cohort studies

**DOI:** 10.18632/oncotarget.8627

**Published:** 2016-04-07

**Authors:** Yong Gan, Jiang Wu, Shengchao Zhang, Liqing Li, Shiyi Cao, Naomie Mkandawire, Kun Ji, Chulani Herath, Chao Gao, Hong Xu, Yanfeng Zhou, Xingyue Song, Shanquan Chen, Yawen Chen, Tingting Yang, Jing Li, Yan Qiao, Sai Hu, Xiaoxv Yin, Zuxun Lu

**Affiliations:** ^1^ Department of Social Medicine and Health Management, School of Public Health, Tongji Medical College, Huazhong University of Science and Technology, Wuhan, Hubei, China; ^2^ Bao’an Central Hospital of Shenzhen, Shenzhen, Guangdong, China; ^3^ Department of Management, School of Economics and Management, Jiangxi Science and Technology Normal University, Nanchang, Jiangxi, China; ^4^ Department of Pathophysiology, Shenyang Medical College, Shenyang, Liaoning, China; ^5^ National Institute for Nutrition and Health, Chinese Center for Disease Control and Prevention, Beijing, Changping, China; ^6^ Division of Health System, Policy and Management, JC School of Public Health and Primary Care, The Chinese University of Hong Kong, Sha Tin, Hong Kong SAR, China

**Keywords:** coffee, colorectal cancer, prospective cohort, meta-analysis, epidemiology

## Abstract

A meta-analysis was performed to assess the association of coffee consumption with colorectal cancer and to investigate the shape of the association. Relevant prospective cohort studies were identified by a comprehensive search of the PubMed, Embase and Web of Science databases from their inception through August 2015. Either a random-effects model or fixed-effects model was used to compute the pooled risk estimates when appropriate. Linear and nonlinear dose-response meta-analyses were also performed. Nineteen prospective cohort studies involving 2,046,575 participants and 22,629 patients with colorectal cancer were included. The risk of colon cancer was decreased by 7% for every 4 cups per day of coffee (RR=0.93, 95%CI, 0.88-0.99; *P*=0.199). There was a threshold approximately five cups of coffee per day, and the inverse association for colorectal cancer appeared to be stronger at a higher range of intake. However, a nonlinear association of rectal cancer with coffee consumption was not observed (*P* for nonlinearity = 0.214). In conclusion, coffee consumption is significantly associated with a decreased risk of colorectal cancer at ≥ 5 cups per day of coffee consumption. The findings support the recommendations of including coffee as a healthy beverage for the prevention of colorectal cancer.

## INTRODUCTION

Colorectal cancer is the third most commonly diagnosed cancer in males and the second in females, with an estimated 1.4 million cases and 693,900 deaths occurring in 2012 worldwide [1]. Ecological and migrant studies have provided strong evidence that environmental factors, including lifestyle and dietary factors, are associated with colorectal cancer risk [2–5]. It has been proposed that the risks associated with coffee consumption could be explained by markers of inflammation or insulin resistance, which have been positively associated with colorectal cancer [6–8]. Therefore, the primary prevention of colorectal cancer worldwide is a considerable priority in public health and clinical medicine [5].

Certain foods and drinks, including coffee, have been implicated in the etiology of colorectal carcinoma [9, 10]. Coffee is one of the most commonly consumed beverages worldwide, with an average consumption of 1.1 kg per capita and 4.5 kg per capita in high- income countries [11]. Thus, investigating the potential role of coffee consumption in the etiology of colorectal cancer is an important public health concern.

In a previous meta-analysis conducted by Li et al. [12], the association between coffee consumption and the risk of colorectal cancer was significant in case-control studies, but it was not significant in cohort studies. Initially, the meta-analysis included only 16 studies, but since then, more cohort studies have been published with inconsistent results. Moreover, the meta-analysis did not investigate the dose-response relationship. Importantly, several issues emerged from the inconsistent results of later studies that still warranted further investigation, including whether it was coffee consumption that prevented the risk of colorectal cancer according to the anatomical subsite, whether the associations were consistent among different subtypes of coffee, and which levels of consumption of coffee had the greatest protection. To investigate these key issues, we conducted a meta-analysis of prospective cohort studies to investigate the association between high and low coffee consumption levels and colorectal cancer risk and to quantify the dose-response relationship between coffee consumption and colorectal cancer risk.

## RESULTS

### Literature search

**Figure 1:**
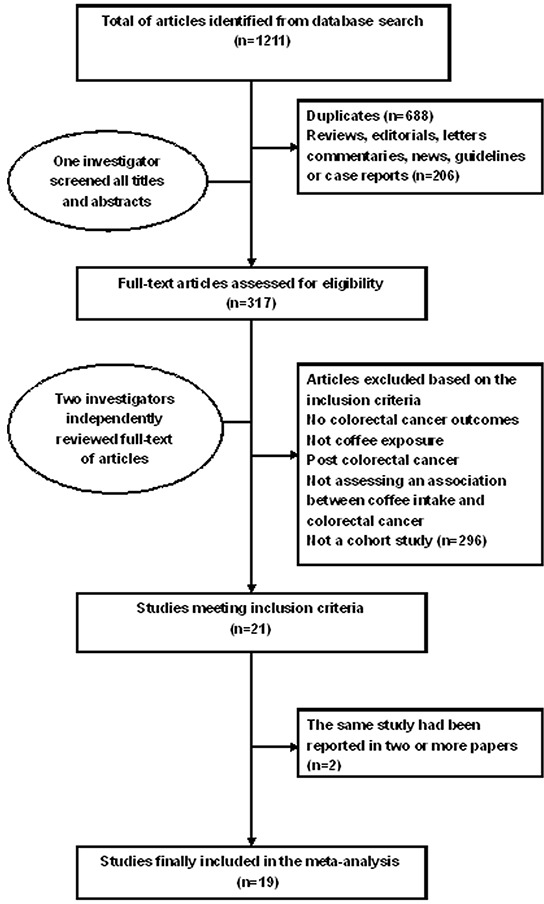
Screening and selection process of studies investigating effect of coffee consumption on colorectal cancer

The search identified 1211 articles from the PubMed, Embase, and Web of Science databases. After the initial screening, based on the titles and abstracts, 317 articles remained for further full-text assessment. After retrieving the full-text review for detailed evaluation, 19 prospective cohort studies [13–31] examining the association between coffee consumption and colorectal cancer risk were identified. The results of the literature research and selection are presented in Figure 1.

### Study characteristics

Supplementary Table S1 shows the main characteristics of the included studies published between 1986 and 2014. Nineteen cohort studies were included in the analysis of the highest versus the lowest coffee intake and colorectal cancer risk, and 17 of these studies were included in the dose-response analysis. Nine studies were from Europe, five from the United States, and five from Asia. The numbers of participants in each study ranged from 11,644 to 489,706, with follow-up durations ranging from 4.5 to 18 years, comprising 2,046,575 individuals and 22,629 cases of colorectal cancer. The median intake of coffee across categories within each study ranged from 0 to 12.5 cups per day. Four studies assessed the coffee consumption without using a specific dietary assessment method, and the rest of studies assessed coffee consumption with food frequency questionnaires (FFQ). Three studies investigated the associations of both caffeinated and decaffeinated coffee consumption with colorectal cancer risk [19, 28, 31]. Four studies assessed the association of coffee consumption with colon cancer risk by anatomical sites [18, 27, 28, 31]. The mean Newcastle-Ottawa Scale (NOS) score was 7.6, suggesting the high quality of studies included in the meta-analysis.

### Association between coffee consumption and the risk of colorectal cancer

**Figure 2:**
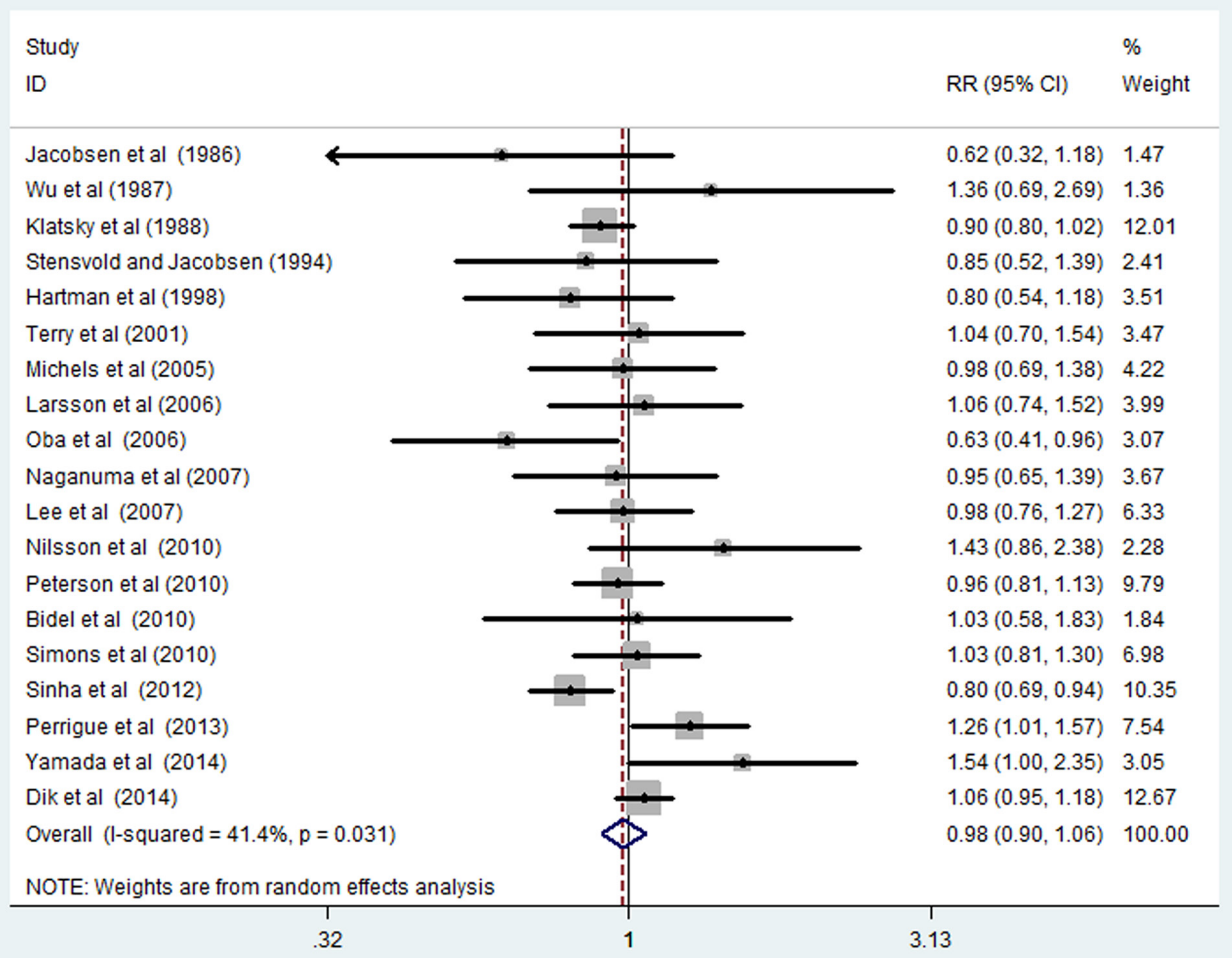
Pooled random effects relative risk (95% CI) of colorectal cancer comparing highest with lowest coffee consumption levels

Nineteen cohort studies [13–31] investigated the relationship between the highest versus the lowest categories of coffee consumption levels and colorectal cancer risk. The random effect summary of relative risk (RR) for the highest versus the lowest coffee consumption categories are shown in Figure 2. The pooled RR of colorectal cancer was 0.98 (95% CI, 0.90–1.06), and a moderate heterogeneity was observed (*I^2^*=41.40%; *P*=0.031).

**Figure 3:**
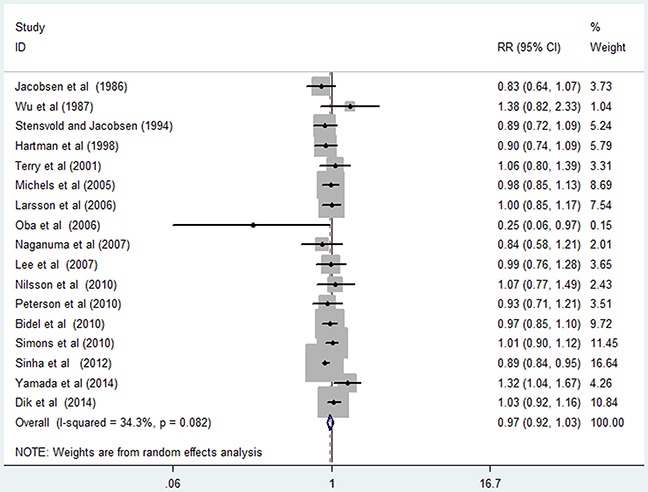
Risk of colorectal cancer associated with per 4 cups/day in coffee consumption

**Figure 4:**
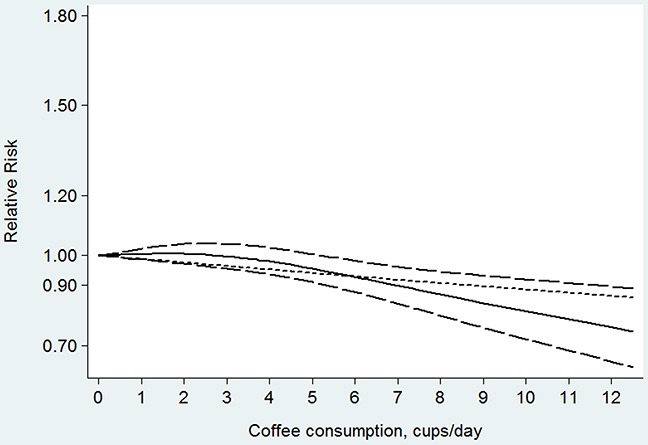
Dose-response relation plots between coffee consumption (cup/day) and the risk of colorectal cancer

Seventeen studies [13, 14, 16–28, 30, 31] were included in the dose-response analysis of coffee consumption with colorectal cancer risk. The summary RR was 0.97 (95% CI, 0.92–1.03) for every 4 cups/day of coffee intake, with a moderate heterogeneity (*I^2^*=34.30%; *P*=0.082; Figure 3). In the cubic spline model that included all studies, we found evidence of a nonlinear association between coffee consumption and the risk of colorectal cancer (Figure 4; *P* for nonlinearity=0.004), without a statistically significant association below 5 cups/day. Compared with people who had no daily consumption of coffee, the RR of colorectal cancer directly estimated from the cubic spline model was 1.00 (95% CI, 0.99–1.02) for 1 cup/day, 1.00 (95% CI, 0.97–1.04) for 2 cups/day, 1.00 (95% CI, 0.96–1.04) for 3 cups/day, 0.98 (95% CI, 0.94–1.03) for 4 cups/day, 0.96 (95% CI, 0.91–1.00) for 5 cups/ day, 0.93 (95% CI, 0.89–0.99) for 6 cups/day, 0.90 (95% CI, 0.85–0.97) for 7 cups/day, and 0.87(95% CI, 0.80–0.95) for 8 cups/day.

### Association between coffee consumption and the risk of colon cancer

**Figure 5:**
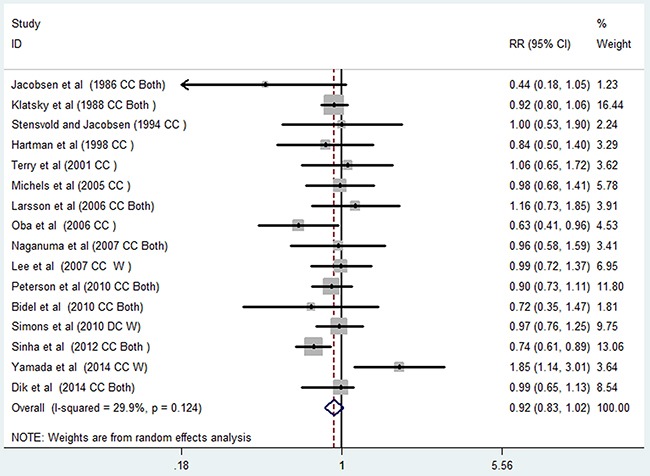
Pooled random effects relative risk (95% CI) of colon cancer comparing highest with lowest coffee consumption levels

Sixteen studies [13, 15–23, 25–28, 30, 31] investigated the relationship between high and low coffee consumption levels and colon cancer risk. The random effect summary of RR for the highest versus the lowest coffee consumption categories are shown in Figure 5. The pooled RR of colon cancer was 0.92 (95%CI, 0.83–1.02), with a low heterogeneity among studies (*I^2^*=29.90%; *P*=0.124).

**Figure 6:**
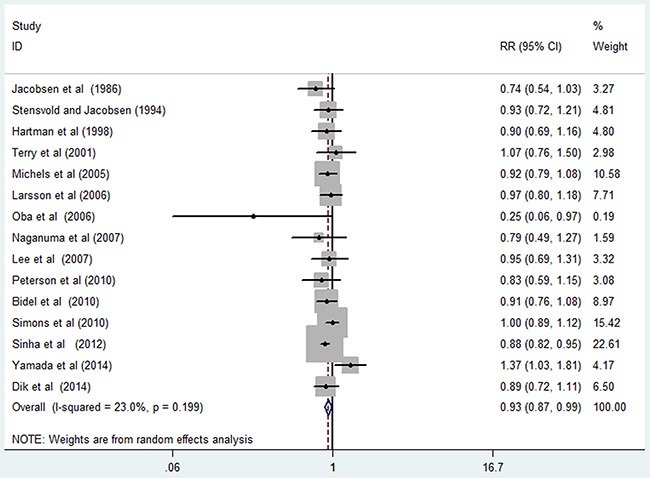
Risk of colon cancer associated with per 4 cups/day in coffee consumption

**Figure 7:**
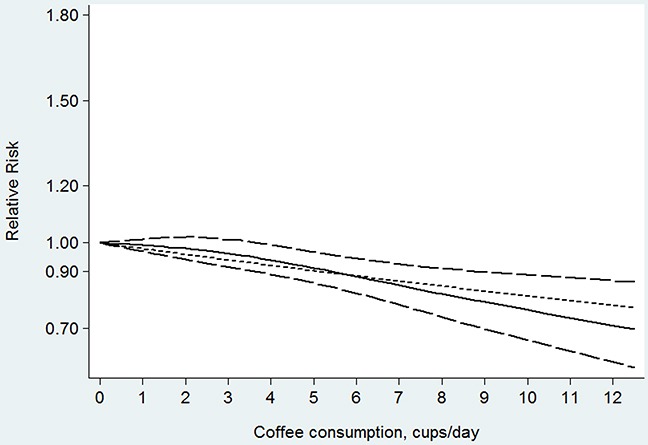
Dose-response relation plots between coffee consumption (cup/day) and the risk of colon cancer

Fifteen studies [13, 16–23, 25–28, 30, 31] were included in the dose-response analysis of coffee consumption with colon cancer risk. The summary RR was 0.93 (95% CI, 0.87–0.99) for every 4 cups/day of coffee intake, with a low heterogeneity (*I^2^*=23.00%; *P*=0.199; Figure 6). In the cubic spline model that included all studies, a nonlinear association between coffee consumption and the risk of colon cancer was observed (Figure 7; *P* for nonlinearity <0.001). Compared to people who had no coffee consumption, the RR of colon cancer estimated directly from the cubic spline model was 0.99 (95% CI, 0.97–1.01) for 1 cup/day, 0.98 (95% CI, 0.95–1.02) for 2 cups/day, 0.97 (95% CI, 0.92–1.02) for 3 cups/day, 0.94 (95% CI, 0.89–0.99) for 4 cups/day, 0.91 (95% CI, 0.86–0.97) for 5 cups/day, 0.89 (95% CI, 0.82–0.95) for 6 cups/day, 0.86 (95% CI, 0.78–0.93) for 7 cups/day, and 0.82 (95% CI, 0.74–0.91) for 8 cups/day.

### Association between coffee consumption and the risk of rectal cancer

Fifteen studies [13, 15–20, 22, 23, 25–28, 30, 31] were included in the analysis of high versus low intake of coffee and rectal cancer risk. The pooled RR of rectal cancer was 1.06 (95% CI, 0.95–1.19), with a low heterogeneity (*I^2^*=13.00%; *P*=0.308; Supplemental Figure S1).

Fourteen studies [13, 16–20, 22, 23, 25–28, 30, 31] were included in the dose-response analysis of coffee consumption with rectal cancer risk. The summary RR was 1.05 (95% CI, 0.97–1.13) for every 4 cups/day of coffee intake, with a low heterogeneity among studies (*I^2^*=11.00%; *P*=0.333; Supplemental Figure S2). In the cubic spline model that included all studies, a nonlinear association between coffee consumption and the risk of rectal cancer was not observed (Supplemental Figure S3; *P* for nonlinearity=0.222).

### Subgroup and sensitivity analyses

The results from subgroup analyses that examined the robustness of the primary results and explored the sources of potential heterogeneity are shown in Supplementary Figure S2. No statistically significant sources of heterogeneity was identified for the association between high versus low coffee consumption and the risk of colorectal cancer in the meta-regression analysis of sex, study location, cancer subsites, duration of follow-up, specific dietary assessment method, body mass index (BMI), smoking, alcohol, physical activity, dairy products/calcium intake, energy intake, folate intake, and consumption of red and processed meat (*P* > 0.05 for each). Importantly, we found that among studies published before 2000, coffee consumption was significantly associated with the risk of colorectal cancer (RR: 0.89, 95% CI, 0.80, 0.99), but in later studies, coffee consumption was not significantly associated with colorectal cancer risk (RR: 1.00, 95% CI, 0.94, 1.07). According to stratified analyses by the subtypes of coffee, a significant inverse association between decaffeinated coffee consumption and colorectal cancer risk was identified (RR: 0.89, 95% CI, 0.80, 0.99), and caffeinated coffee consumption was not significantly associated with colorectal cancer risk (RR: 0.99, 95% CI, 0.90, 1.10).

Findings with fixed-effects inverse-variance weighting were similar for colorectal (RR: 0.97; 95% CI: 0.92, 1.03) and rectal cancer (RR: 1.07; 95% CI: 0.97, 1.18) in the analysis of high versus low intake of coffee. In contrast, with fixed-effects inverse-variance weighting, coffee consumption was inversely associated with colon cancer (RR: 0.91; 95% CI: 0.84, 0.98).

In the sensitivity analysis of high versus low coffee intake, omitting one study in turn, no particular study explained the results found for colorectal cancer; the pooled RR ranged from 0.96 (95% CI, 0.89–1.04; *I^2^*=32.40%, *P*=0.091) to 1.00 (95% CI, 0.93–1.09; *I^2^*=28.10%, *P*=0.130). Similarly, in the dose-response analysis, the non-significant inverse association of coffee intake with colorectal cancer was still unchanged by removing one study at a time, with a pooled RR ranging from 0.95 (95% CI, 0.91–1.00; *I^2^*=11.20%, *P*=0.325) to 0.99 (95% CI, 0.94–1.04; *I^2^*=11.00%, *P*=0.327).

In addition, the effect of excluding studies from the dose-response analysis on the results was explored. When the analysis of high versus low coffee intake was restricted to the studies that were included in the dose-response analysis, the summary RR was 0.97 (95% CI, 0.89–1.06, *I^2^*=32.90%, *P*=0.093 for heterogeneity), similar to the original analysis including all studies.

The lowest reference category according to one study by Hartman et al. [17] was less than or equal to 4 cups/day, which was higher than the highest exposure category of six studies. With the systematic removal of this study, the pooled RR for colorectal cancer for the highest versus the lowest categories of coffee intake did not change. The summary RR was 0.99 (95% CI, 0.91–1.07; *I^2^*=42.80%, *P*=0.028) after the exclusion of this study. In the dose-response analysis, when this study was excluded, the nonlinear associations of colorectal and colon cancers with coffee consumption did not change (*P* for nonlinearity =0.007 and *P* for nonlinearity =0.001, respectively) when this study was excluded. Similar results were found for an increment of 4 cups/day coffee consumption and the risk of colorectal, colon and rectal cancers after the removal of this study. Thus, it is relatively appropriate to include this study in our dose-response analysis.

### Publication bias

In the analyses of the highest compared with the lowest category, the Begg's and the Egger's tests did not provide any significant evidence of publication bias for studies that investigated the relationship between coffee consumption and colorectal cancer (Begg's *P*=0.944, Egger's *P*=0.764), colon cancer (Begg's *P*=0.620, Egger's *P*=0.699), and rectal cancer (Begg's *P*=1.000, Egger's *P*=0.822). Additionally, no significant evidence of substantial publication bias was observed for the dose-response analyses (Begg's *P*=0.773, Egger's *P*=0.434 for colorectal cancer, Begg's *P*=0.322, Egger's *P*=0.858 for colon cancer, and Begg's *P*=0.827, Egger's *P*=0.384 for rectal cancer).

## DISCUSSION

This meta-analysis showed that coffee consumption was not inversely associated with colorectal, colon, or rectal cancers in the high versus low comparison. In the linear dose-response analysis, we showed that the consumption of 4 cups of coffee per day was associated with a 7% lower risk of colon cancer but was not significantly associated with rectal cancer. There was some evidence of a nonlinear association between coffee consumption and colorectal and colon cancers, with the strongest reduction in risk at higher level of intake.

Three previous studies published in 2009 [32] and 2013 [12, 33] investigated the evidence of coffee consumption's impact on colorectal cancer risk. The results of this updated meta-analysis generally concur and further complement the findings of previous reviews in several important aspects. Je et al. [32] reported that no association was observed between coffee consumption and colorectal cancer risk. However, our meta-analysis included 7 additional cohort studies with larger sample sizes and many more cases, which significantly increased our statistical power to detect potential associations between coffee consumption and colorectal cancer. Additionally, Je et al. did not investigate additional subgroups other than gender, study location and cancer site and did not conduct a dose-response analysis. Li et al. [12] found that coffee consumption was significantly associated with colorectal cancer in case-control studies, but a significant association was not found in cohort studies. Additionally, the nonlinear dose-response association between coffee intake and colorectal cancer was not investigated in the meta-analysis. Tian et al. [33] reported a significant association between coffee consumption and a decreased risk of colorectal cancer among individuals consuming ≥ 4 cups of coffee per day, which was consistent with our results. However, 7 studies were not included, which may have underestimated the effect size. In addition, our review performed more detailed subgroup analyses (such as publication year, subtypes of coffee, adjustment for physical activity, and intake of dairy products/calcium and energy) to test the robustness of the results and explore the potential heterogeneity. More importantly, compared with previous reviews, a nonlinear association between coffee consumption and colorectal cancer was observed in our meta-analysis.

The nonlinear relationship between coffee consumption and colorectal and colon cancer risk might also be true on the basis of plausible biological mechanisms. Coffee is a complex mixture of more than a thousand compounds. Some constituents (such as very small amounts of aromatic hydrocarbons and heterocyclic amines) have been described as having genotoxic and mutagenic properties. For example, coffee contains acrylamide and caffeine, which have potential carcinogenic effects [34, 35]. In addition, coffee consumption might be been associated with higher insulin sensitivity and a lower risk of type 2 diabetes mellitus [36], which is a known risk factor for colorectal cancer. Conversely, coffee also contains many bioactive compounds, including phenolic acids with strong antioxidant properties and cafestol and kahweol with anticarcinogenic activity [37, 38]. Thus, the nonlinear relationship might be due to a combination of detrimental and beneficial effects. Our findings showed that coffee consumption had some degree of a protective effect on the incidence of colorectal or colon cancer. For heavy coffee (≥5 cups/day) consumption, beneficial effects may be greater than adverse effects, and the inverse association of higher coffee intake and colorectal cancer became stronger.

We observed a significant inverse association between coffee consumption and colorectal cancer risk in earlier publications (2000 or earlier). One potential interpretation of this finding was that coffee brewing methods had changed over time, and currently, the filter method is more popular, effectively replacing unfiltered forms of coffee, such as boiled coffee, which were more widely consumed by participants in earlier studies [39]. Boiled (unfiltered) coffee has been reported to contain higher amounts of lipid components (diterpenes, such as cafestol and kahweol) [40, 41]. Cafestol and kahweol may lower colorectal cancer risk by reducing bile acid synthesis and secretion [42–44], and inhibiting the activity of CYP1A2 and NAT2 [45, 46]. To date, only a cohort study from Sweden separately investigated the role of filtered and boiled coffee on colorectal cancer risk but did not identify a significant association [24]. The brewing method needs to be considered in prospective cohort studies of coffee consumption and colorectal cancer.

A significant inverse association with colorectal cancer risk was observed with decaffeinated coffee consumption, but not with caffeinated coffee consumption, which is an interesting phenomenon. There were several potential explanations. The differences could reflect chance or perhaps residual confounding, if the lifestyles differed between individuals who regularly drank decaffeinated coffee and those who drank caffeinated coffee. Previous studies showed that participants who drank decaffeinated coffee tended to drink less alcohol, eat more fruit and vegetables, and consume less red meat than participants who drank caffeinated coffee [19, 28]. Another possible explanation was that the compounds in decaffeinated coffee, including, the lack of caffeine, were beneficial in cancer prevention. More studies investigating the potential difference between the different subtypes of coffee are warranted.

This meta-analysis has several strengths. Because we based the analysis on prospective studies, the findings are unlikely to be explained by recall and selection biases. Our meta-analysis included a large number of studies and more than 22,600 cases, with over two million participants in the coffee consumption analysis. Thus, we had adequate statistical power to analyze the association of coffee consumption with colorectal cancer risk, clarify the shape of the dose-response relationship between coffee consumption and the risk of colorectal cancer, and to detect low to moderate reductions in risk. We quantified the association between coffee consumption and the risk of colorectal cancer by carrying out linear and nonlinear dose-response analyses and found a significant nonlinear association of coffee consumption with colorectal and colon cancer risk.

Several limitations should be acknowledged. Most original studies used FFQ to assess levels of coffee consumption. Although validation studies showed that FFQ was a reasonable tool to assess the intakes of coffee, measurement error was inevitable. Second, although the original studies included in our analysis were adjusted for multiple major risk factors for colorectal cancer except for three studies, the possibility of residual confounding by imprecisely or unmeasured confounders should be considered, as coffee consumers tended to follow less healthy behaviors, including lower levels of physical activity, higher prevalence of smoking and overweight/obesity, and higher intake of alcohol and red and processed meat [19, 22, 32]. Many but not all of the studies were adjusted for potential confounding factors, although not all potential confounders were adjusted in every study. In analyses stratified by adjustment for confounding factors, however, we found that the association between coffee intake and the risk of colorectal cancer persisted in most subgroups. Thus, it was difficult to identify the independent effects of coffee consumption from those of unhealthy diet and lifestyle in observational analyses. Third, our meta-analysis did not consider the differences between types of coffee bean, brewing methods, and serving sizes for coffee among original studies. Final, although publication bias can be a problem in meta-analyses of published studies, we did not find evidence of such a bias in this analysis.

In summary, this meta-analysis suggests a nonlinear association between coffee intake and colorectal cancer. There was a threshold approximately 5 cups of coffee consumed per day and further reductions in the risk of colorectal cancer with higher intake. Studies with larger sample sizes and longer follow-up times are warranted to confirm our results. Given that coffee is consumed very commonly and the levels of morbidity and mortality of colorectal cancer are high worldwide, the results of our study provide practical and valuable insight into the prevention of colorectal cancer and the study of its etiology.

## MATERIALS AND METHODS

### Search strategy

This systematic review was conducted following the Meta-analysis of Observational Studies in Epidemiology (MOOSE) guidelines [47]. We performed a comprehensive search of the PubMed, Embase and Web of Science databases from their inception through August 2015 for prospective cohort studies published in peer-reviewed journals that described an association between coffee consumption and the risk of colorectal cancer. We used “coffee” OR “caffeine” OR “decaffeinated” OR “dietary intake” OR “beverages” and “colorectal” OR “colon” OR “rectal” OR “rectum” OR “bowel” combined with “cancer” OR “carcinoma” OR “tumor” OR “neoplasm” and “cohort studies” OR “prospective studies” OR “follow-up studies” as the search terms. The search was restricted to human studies. No restrictions were imposed on the language of publications. Abstracts, non-original papers (reviews, editorials, or letters), grey literature, and unpublished results or information were not included. We also reviewed the reference lists of all included original studies and the reference lists of the published systematic reviews [12, 32]. One investigator (Y. G.) screened the titles and abstracts of all identified articles; two investigators (Y. G and L.Q. L.) reviewed the eligibility of potentially relevant full-text articles.

### Study selection

Studies were included in this meta-analysis according to the following criteria: (1) the study was a prospective cohort study, including nested case-control studies with a prospective design; (2) the exposure of interest was coffee consumption, including total coffee, caffeinated coffee, or decaffeinated coffee; (3) the outcome of interest was the risk of colorectal cancer; (4) the participants were free of colorectal cancer at baseline; and (5) the study reported risk estimates with corresponding 95% CI for the association between coffee and colorectal cancer or provided corresponding data to calculate the variance. In cases where multiple publications from the same study were available, we included the publication that presented the results with sufficient detail to be incorporated into dose-response analyses or the publication with the largest number of cases.

### Data extraction

Two investigators (Y. G. and L. Q. L.) independently extracted the data from each study using a standardized electronic format, including the last name of the first author, publication year, study location, sample size, sex, age range or mean age at entry, length of follow-up, number of cases, method of assessment of exposure, outcome measurements, RRs with corresponding 95% CIs for all categories of coffee consumption, and covariates adjusted for in the multivariable analysis. We extracted risk estimates with the most adjustment (when available). If a study only reported caffeinated coffee consumption instead of total coffee consumption, caffeinated coffee consumption was also included in the total coffee consumption analysis. For dose-response analysis, when studies reported the consumption in milliliters per day or week or month, we standardized all the data into cups per day; 237 ml of coffee was equal to 1 cup. Differences in data extraction between the two investigators were resolved by discussion with the third investigator (Z. X. L.).

### Quality assessment

Quality assessment was performed according to the NOS [48]. The scale was a nine-point scale that allocated points on the basis of the process of selection of the cohorts (0-4 points), the comparability of cohorts (0-2 points), and the identification of outcomes (0-3 points). We assigned scores of 0-3, 4-6, and 7-9 for the low, moderate, and high quality of studies, respectively. Each study was rated independently by two investigators (Y. G. and L.Q. L.).

### Statistical analysis

RRs were considered as the common measure of the association between coffee consumption and colorectal cancer risk, and the hazard ratios (HRs) were considered to be equivalent to RRs. We preferentially pooled multivariable adjusted RR estimates where such estimates were available. When the adjusted estimates were unavailable (three studies), we pooled the unadjusted estimates. A random effects model was used to calculate the summary RRs and 95% CIs for the highest versus lowest level of consumption of coffee and for the dose-response analysis. We compared these findings to the fixed-effects inverse-variance weights in sensitivity analyses. For studies that reported results separately for colon and rectal cancer or for men and women, we combined the estimates using a fixed-effects model to obtain an overall estimate for colorectal cancer or both sexes combined in the primary meta-analysis [49].

For the dose-response analysis, we used the previously described method [50] to calculate the trend from the correlated estimates for the log relative risk across categories of coffee consumption. The amount of coffee consumption, the distributions of cases and person years, and RRs and 95% CI were extracted according to the method. If the person years were not available for each category of coffee intake but the study reported the total number of cases/person-years, we estimated the distribution. If the consumption of coffee was analyzed by quartiles (and could be approximated), the total number of person years was divided by 4 when the data were analyzed by quartiles to derive the number of person-years in each quartile. If this information was missing and the results were reported by functional categories (e.g., <1, 1–2, 3–5, and≥6 cups per day, we used variance-weighted least squares regression to estimate the slopes [51]. The median or mean coffee consumption in each category was assigned to the corresponding dose of consumption. The midpoint of the upper and lower boundaries was considered to be the dose of each category if the median or the mean intake per category was unavailable. When the lower boundary for the lowest category was not provided, the assigned median value was half of the upper boundary of that category. If the highest category was open-ended, we assumed that the median value of the category was the cut-off point plus a 25% increase.

We presented the dose-response results in the forest plots for an increase of 4 cups per day. We also examined the potential non-linear associations between coffee consumption and colorectal cancer risk using the two-stage random-effects dose-response meta-analysis. This was conducted by modeling consumption with the use of restricted cubic splines with 3 knots at fixed percentiles (10%, 50%, and 90%) of the distribution [52]. A *P* value for nonlinearity was calculated by testing that the coefficient of the second spline transformation was equal to zero against the null hypothesis [53].

Statistical heterogeneity across studies was assessed using the *I^2^* statistic (ranging from 0% to 100%). *I^2^* values of 25%, 50%, and 75% represented cut-off points for low, moderate, and high heterogeneity, respectively [54].

Subgroup and meta-regression analyses by sex, study location, cancer subsite, duration of follow-up, specific dietary assessment method, subtypes of coffee, publication year, and adjustment for confounding factors such as BMI, smoking, alcohol, physical activity, dairy products/calcium intake, energy intake, fruit and vegetable intake, folate intake and red and the consumption of processed meat were conducted to investigate the potential sources of heterogeneity among studies. Sensitivity analyses were performed to evaluate the effect of removing a single study from the analysis on the pooled risk estimates.

The Begg's test [55] and the Egger's test [56] were used to assess the potential publication bias, with the results indicating publication bias when *P* < 0.10. All statistical analyses were conducted with STATA version 12.0 (StataCorp, College Station, Texas, USA). *P* values were two-tailed with a significance level of 0.05, except where otherwise specified.

## SUPPLEMENTARY MATERIALS FIGURES AND TABLES






