# Supplementary material for: Association of coffee consumption with risk of colorectal cancer: a meta-analysis of prospective cohort studies
Source: Oncotarget. 2016 Apr 7;8(12):18699–711. doi: 10.18632/oncotarget.8627 (PMC5386640; doi:10.18632/oncotarget.8627)
Supplement: Supplementary file 1 [file oncotarget-08-18699-s001.pdf]

SUPPLEMENTARY FIGURES AND TABLES

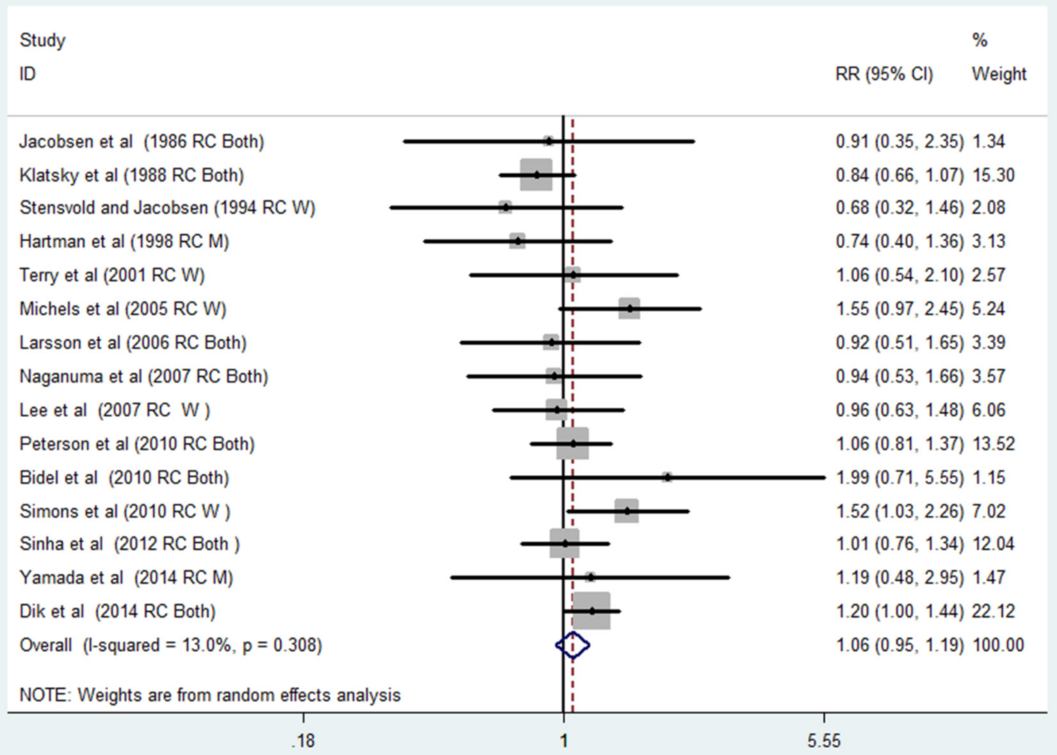

Supplementary Figure S1: Pooled random effects relative risk (95% CI) of rectal cancer comparing highest with lowest coffee consumption levels.

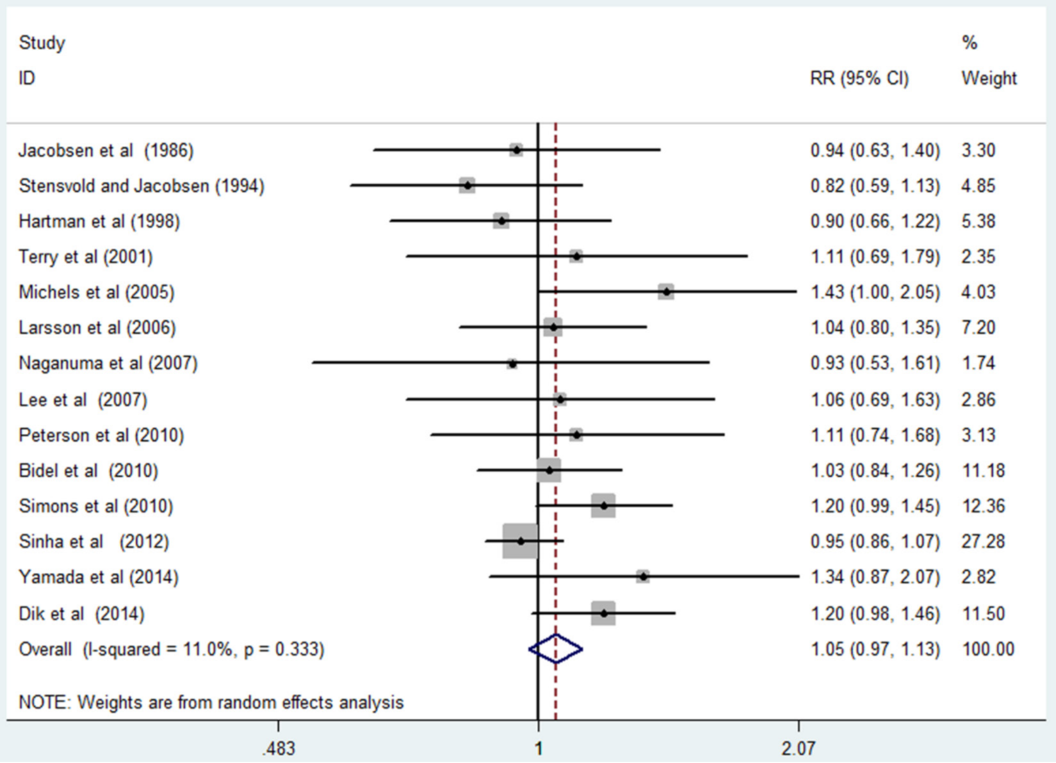

Supplementary Figure S2: Risk of rectal cancer associated with per 4 cups/day in coffee consumption.

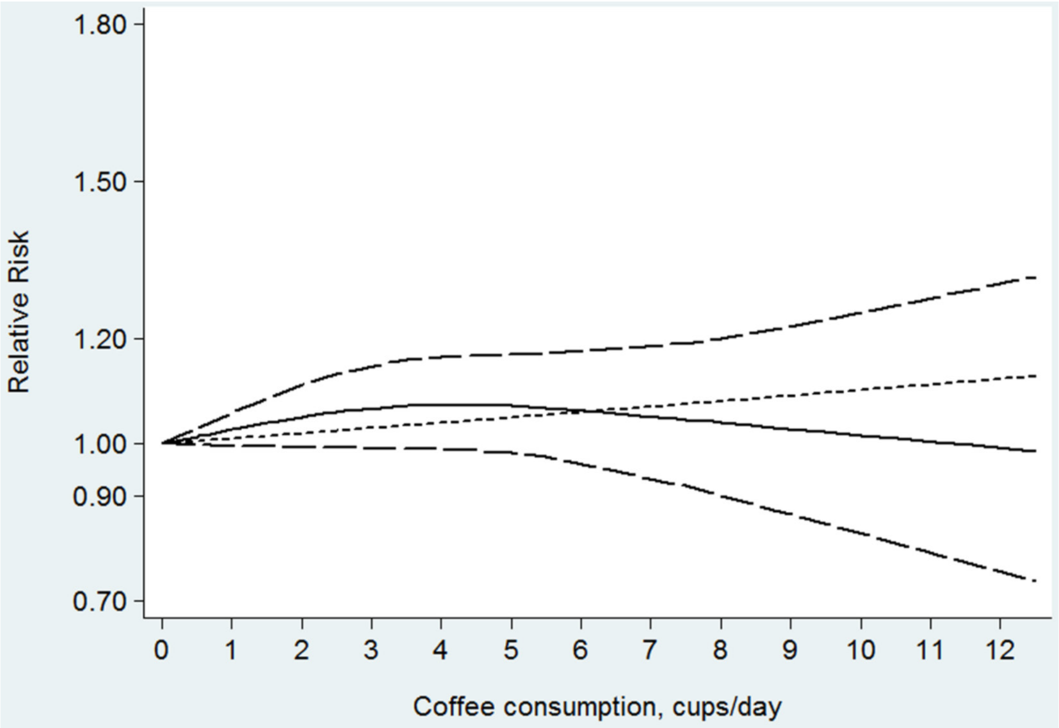

Supplementary Figure S3: Dose-response relation plots between coffee consumption (cup/day) and the risk of rectal cancer.

Supplementary Table S1: Characteristics of studies included in the meta-analysis of coffee consumption in relation to risk of colorectal cancer.

See Supplementary File 1

Supplementary Table S2: Subgroup analyses of coffee consumption and risk of colorectal cancer (highest versus lowest category).

See Supplementary File 2
