# Supplementary material for: Association of coffee consumption with risk of colorectal cancer: a meta-analysis of prospective cohort studies
Source: Oncotarget. 2016 Apr 7;8(12):18699–711. doi: 10.18632/oncotarget.8627 (PMC5386640; doi:10.18632/oncotarget.8627)
Supplement: Supplementary file 2 [file oncotarget-08-18699-s002.docx]

**Supplemental Material**

**Supplemental Table 1 Characteristics of studies included in the meta-analysis of coffee consumption in relation to risk of colorectal cancer**

| **Study source** | **Sex** | **Follow-up (years)*** | **Age at baseline (years)** | **No of**  **participants** | **No of case** | **Exposure**  **assessment** | **Outcome ascertainment** | **Coffee categories (highest vs. lowest)** | **Relative risk (95%CI)** | **Adjustment for covariates** |
| --- | --- | --- | --- | --- | --- | --- | --- | --- | --- | --- |
| Jacobsen et al, 1986, Norway | M/F | 11.5 | Range 35+ | 16,555 | 97 CC 63 RC | Nonspecific diet questionnaire | Cancer Registry of Norway | CC: ≥7 cups/d vs.<2cups/d RC: ≥7 cups/d vs.<2cups/d | 0.44 (0.18 to 1.05) 0.91 (0.35 to 2.35) | No covariate adjustment |
| Wu et al,1987, United States | M/F | 4.5 | Range 35+ | 11,644 | 126 CRC | Nonspecific diet questionnaire | Confirmed  cases | CRC, M: ≥4 cups/d vs. ≤1cup/d CRC, F: ≥7 cups/d vs.≤1cup/d | 1.54 (0.60 to 3.70) 1.17 (0.40 to 3.10) | Age |
| Klatsky et al,1988, United States | M/F | 6 | Range NA | 106,203 | 203 CC 66 RC | Nonspecific diet  questionnaire | Medical records and confirmed  cases | CC: Per cup increase of  coffee consumption RC: Per cup increase of  coffee consumption | 0.92 (0.80 to 1.06) 0.84 (0.66 to 1.07) | Age, sex, alcohol, smoking, BMI, race, education, and serum cholesterol |
| Stensvold and Jacobsen, 1994, Norway | M/F | 10.1 | Range 35-54 | 42,973 | 130 CC 79 RC | FFQ | Norwegian  Cancer Registry | CC, M: ≥7 cups/d vs. ≤2cups/d CC, F: ≥7 cups/d vs. ≤2cups/d RC, M: ≥7 cups/d vs. ≤2cups/d RC, F: ≥7 cups/d vs. ≤2cups/d | 1.00 (0.44 to 2.29) 1.00 (0.36 to 2.74) 0.69 (0.29 to 1.66) 0.64 (0.13 to 3.19) | No covariate adjustment |
| Hartman et al,1998, Finland | M | 8 | Range 50-69 | 27,111 | 106 CC 79 RC | FFQ | Finnish Cancer Registry | CC: >6 cups/d vs. ≤4cups/d RC: >6 cups/d vs. ≤ 4cups/d | 0.84 (0.50 to 1.40) 0.74 (0.40 to 1.36) | Age, BMI, physical activity, intervention group, serum cholesterol for RC, calcium, and tea for RC |
| Terry et al, 2001, Sweden | F | 9.6 | Range 40-74 | 61,463 | 460CRC 291CC 159RC | FFQ | Swedish Cancer Registry | CRC: ≥4 cups/d vs. < 1cup/d CC: ≥4 cups/d vs. < 1cup/d RC:≥4 cups/d vs. < 1cup/d | 1.04 (0.70 to 1.54) 1.06 (0.65 to 1.72) 1.06 (0.54 to 2.10) | Age, alcohol, BMI, education, calories, red meat, fat, fiber, calcium, folic acid, vitamin C, and vitamin D |
| Michels et al,2005, United States | M | 12 | Range 40-75 | 46,099 | 552 CRC 446 CC 106 RC | FFQ | National  Death Index and medical records | Caffeinated coffee CRC: >5 cups/d vs. 0 cup/d Decaffeinated coffee CRC: ≥2 cups/d vs. 0 cup/d Caffeinated coffee CC: >5 cups/d vs. 0 cup/d Decaffeinated coffee CC: ≥2 cups/d vs. 0 cup/d Caffeinated coffee RC: ≥4 cups/d vs. 0 cup/d Decaffeinated coffee RC: ≥2 cups/d vs. 0 cup/d | 1.09 (0.55 to 2.17) 0.85 (0.64 to 1.12) 1.39 (0.69 to 2.78) 0.94 (0.70 to 1.28) 1.33 (0.69 to 2.56) 0.50 (0.20 to 1.27) | Age, BMI, height, alcohol, smoking, Physical activity, family history of CRC, sigmoidoscopy, aspirin use, vitamin supplement, total caloric intake, and red meat |
| Michels et al,2005, United States | F | 18 | Range 30-55 | 87,794 | 886 CRC 731CC 155RC | FFQ | National  Death Index and medical records | Caffeinated coffee CRC: >5 cups/d vs. 0 cup/d Decaffeinated coffee CRC: ≥2 cups/d vs. 0 cup/d Caffeinated coffee CC: >5 cups/d vs. 0 cup/d Decaffeinated coffee CC: ≥2 cups/d vs. 0 cup/d Caffeinated coffee RC: ≥4 cups/d vs. 1 cup/d Decaffeinated coffee RC: ≥2 cups/d vs. 0 cup/d | 0.94 (0.63 to 1.40) 0.80 (0.61 to 1.04) 0.85 (0.55 to 1.32) 0.90 (0.67 to 1.21) 1.80 (0.94 to 3.44) 0.48 (0.26 to 0.89) | Age, BMI, height, alcohol, smoking, physical activity, family history of CRC, sigmoidoscopy, aspirin use, vitamin supplement, total caloric intake, red meat, menopausal status, and postmenopausal hormone use |
| Larsson et al,2006, Sweden | M/F | 6.7 | Range M:45-74  F: 40-76 | 81,922 | 723CRC 469 CC 256 RC | FFQ | Swedish Cancer Register and confirmed cases | CRC: ≥6 cups/d vs. < 1cup/d CC: ≥6 cups/d vs. < 1cup/d RC:≥6 cups/d vs. < 1cup/d | 1.06 (0.74 to 1.52) 1.16 (0.73 to 1.85) 0.92 (0.51 to 1.65) | Age, BMI, smoking, physical activity, family history of CRC and diabetes, aspirin use, multivitamin use, calories, red meat, fruits, vegetables, milk, for women post-menopausal hormone use |
| Oba et al,2006, Japan | M/F | 7 | Range 35+ | 30,221 | 213 CC | FFQ | Cancer registry and Confirmed  cases | CC, M: ≥1 cup/d vs. < 1cup/month CC, F: ≥1 cup/d vs. < 1cup/month | 0.81 (0.46 to 1.42) 0.43 (0.22 to 0.85) | Age, BMI, height, alcohol intake, smoking, physical activity, black and green tea intake |
| Naganuma et al, 2007, Japan | M/F | 11 | Range 40-64 | 38,701 | 457 CRC 281 CC 180 RC | FFQ | Cancer registry and Confirmed  cases | CRC:≥3 cups/d vs. 0 cup/d CC:≥3 cups/d vs. 0 cup/d RC:≥3 cups/d vs. 0 cup/d | 0.95 (0.65 to 1.39) 0.96 (0.58 to 1.59) 0.94 (0.53 to 1.66) | Age, sex, BMI, alcohol, smoking, walking time, family history of CRC, education, calories, fruits, vegetables, meat, black/green tea, for women menopausal status, numbers of pregnancies and deliveries, age at menarche, age at first delivery |
| Lee et al, 2007, Japan | M/F | 10 | M: mean 51.9 W: mean 52.3 | 96,162 | 1163 CRC 763 CC 400 RC | FFQ | Cancer registries and Death  certificates | CRC, M:≥3 cups/d vs. almost never CRC, W:≥3 cups/d vs. almost never CC, M:≥3 cups/d vs. almost never CC, W≥3 cups/d vs. almost never RC, M:≥3 cups/d vs. almost never RC, W:≥3 cups/d vs. almost never | 1.10 (0.82 to 1.47) 0.68 (0.40 to 1.15) 1.15 (0.80 to 1.66) 0.60 (0.31 to 1.19) 1.01 (0.61 to 1.66) 0.84 (0.36 to 1.94) | Age, BMI, alcohol, smoking, physical activity, study area, family history of CRC, beef, pork, green vegetables, black tea, green tea, and Chinese tea |
| Nilsson et al,2010, Sweden | M/F | 6 | Mean 50 | 64,603 | 321CRC | FFQ | Regional cancer registry | CRC:≥4 cups/d vs. < 1cup/d | 1.43 (0.86 to 2.38) | Age, sex, BMI, smoking, education, and recreational physical activity |
| Peterson et al,2010, Singapore | M/F | 9.8 | Range 45–74 | 61,321 | 961 CRC 591 CC 370 RC | FFQ | Singapore Cancer Registry and the Singapore Registry of Births and Deaths | CC: ≥2 cups/d vs. < 1cup/d RC:≥3 cups/d vs. < 1cup/d | 0.90 (0.73 to 1.11) 1.06 (0.81 to 1.37) | Age, sex, dialect group, year of recruitment, education, BMI, smoking, alcohol consumption, physical activity, history of diabetes, family history of CRC, and green tea intake |
| Bidel et al ,2010, Finland | M/F | 18 | Range 26-74 | 60,041 | 538 CRC 304 CC 234 RC | Nonspecific diet questionnaire | Finnish Cancer Registry and confirmed cases | CRC:≥10 cups/d vs. 0 cup/d CC:≥10 cups/d vs. 0 cup/d RC:≥10 cups/d vs. 0 cup/d | 1.03 (0.58 to 1.83) 0.72 (0.35 to 1.47) 1.99 (0.71 to 5.55) | Age, sex, study year, education, cigarette smoking, alcohol consumption, leisure-time  physical activity, history of diabetes, tea consumption and BMI |
| Simons et al,2010, Netherland | M/F | 13.3 | Range 55-69 | 120,852 | 2899 CRC | FFQ | Netherlands  cancer registry and confirmed cases | CRC, M: >6 cups/d vs. ≤2cups/d CRC, W: > 6 cups/d vs. ≤2cups/d PC, M: >6 cups/d vs. ≤2cups/d PC, W: >6 cups/d vs. ≤2cups/d DC, M: >6 cups/d vs. ≤2cups/d DC, W: >6 cups/d vs. ≤2cups/d RC, M: >6 cups/d vs. ≤2cups/d RC, W: > 6 cups/d vs. ≤2cups/d | 1.00 (0.74 to 1.36) 1.07 (0.74 to 1.55) 0.91 (0.58 to 1.44) 1.12 (0.67 to 1.88) 0.93 (0.59 to 1.47) 0.96 (0.20 to 1.80) 1.60 (0.96 to 2.66) 1.41 (0.75 to 2.63) | Age, family history of CRC, no occupational physical activity, smoking, educational, BMI, ethanol, meat, processed meat, folate, vitamin B6, fiber, and fluid intake from other fluids |
| Sinha et al ，2012, United States | M/F | 10.5 | Range 50-71 | 489,706 | 6946 CRC 5072 CC 1874 RC | FFQ | State cancer registries | CRC:≥6 cups/d vs. 0 cup/d CC:≥6 cups/d vs. 0 cup/d RC:≥6cups/d vs. 0 cup/d | 0.80 (0.69 to 0.94) 0.74 (0.61 to 0.89) 1.01 (0.76 to 1.34) | Age , sex, race , education, smoking status , time since quitting for former smokers, smoking dose, ever smoke a pipe or cigar, diabetes , colorectal screening, family history of CRC, regular no steroidal anti-inflammatory drug use , marital status , BMI, frequency of vigorous physical activity , calories , fruit and vegetables , red meat , dietary calcium intake , alcohol , and menopausal hormone therapy in women |
| Perrigue et al , 2013, United States | M/F | 5.8 | Range 50-76 | 67,912 | 323 CRC | FFQ | National Cancer registry and confirmed cases | CRC: ≥1cup/d vs. < 1cup/d | 1.26 (1.01 to 1.57) | No covariate adjustment |
| Yamada et al, 2014, Japan | M/F | 12.7 | Range 40-79 | 58,221 | 1001 CRC 687 CC 314 RC | FFQ | National Cancer registry and confirmed cases | CRC, M:≥4 cups/d vs. < 1cup/d CRC, W:≥4 cups/d vs. < 1cup/d CC, M:≥4 cups/d vs. < 1cup/d CC, W≥4 cups/d vs. < 1cup/d RC, M:≥4 cups/d vs. < 1cup/d RC, W:≥4 cups/d vs. < 1cup/d | 1.57 (0.97 to 2.55) 1.42 (0.57 to 3.50) 1.79 (1.01 to 3.18) 2.02 (0.81 to 5.03) 1.01 (0.61 to 1.66) 1.19 (0.48 to 2.95) 0.00 (---) | Age, smoking, drinking, family history of CRC, education, BMI, walking time, regular meat consumption, and district |
| Dik et al ,2014, Europe | M/F | 11.6 | Range 25-70 | 477,071 | 4234 CRC 2691CC 1543RC | FFQ, recall record | Regional cancer registries and self-report | CRC: >625ml/d vs. ≤ 100ml/d CC: >625ml/d vs. ≤ 100ml/d RC: >625ml/d vs. ≤ 100ml/d | 1.06 (0.95 to 1.18) 0.99 (0.65 to 1.13) 1.20 (1.00 to 1.44) | Age, sex, center, BMI , diabetes mellitus, menopausal status , hormone replacement therapy , physical activity , educational level smoking status, and baseline intake of energy from fat, energy from non-fat, alcohol, fibers, dairy products, red meat and processed meat |

*Mean or median duration of follow-up. Abbreviations: BMI, body mass index; CC, colon cancer; CRC, colorectal cancer; DC; distal colon; F, female; FFQ, food frequency questionnaire; M, male; NA, not available; PC, proximal colon; RC, rectal cancer; SBP, systolic blood pressure.
