# Supplementary material for: Association of coffee consumption with risk of colorectal cancer: a meta-analysis of prospective cohort studies
Source: Oncotarget. 2016 Apr 7;8(12):18699–711. doi: 10.18632/oncotarget.8627 (PMC5386640; doi:10.18632/oncotarget.8627)
Supplement: Supplementary file 3 [file oncotarget-08-18699-s003.docx]

**Supplemental Table 2 Subgroup analyses of coffee consumption and risk of colorectal cancer (highest versus lowest category)***

|  | **No of studies** | **Relative risk** | **(95% CI)** | ***I^2^ (%)*** | ***P* value for heterogeneity** | ***P* value between groups** |
| --- | --- | --- | --- | --- | --- | --- |
| All studies | 19 | 0.97 | 0.92 to 1.03 | 41.4 | 0.031 |  |
| Sex |  | | | | | |
| Men | 10 | 1.01 | 0.88 to 1.17 | 0.00 | 0.766 | 0.85 |
| Women | 10 | 0.94 | 0.79 to 1.12 | 0.00 | 0.508 |  |
| Combined | 8 | 0.97 | 0.91 to 1.03 | 55.3 | 0.013 |  |
| Location |  | | | | | |
| Europe | 9 | 1.03 | 0.95 to 1.12 | 0.00 | 0.602 | 0.91 |
| United States | 5 | 0.92 | 0.85 to 1.00 | 67.9 | 0.014 |  |
| Asia | 5 | 0.97 | 0.86 to 1.09 | 52.8 | 0.075 |  |
| Subsite |  |  |  |  |  |  |
| Colon | 16 | 0.91 | 0.84 to 0.98 | 29.90 | 0.124 | 0.07 |
| Rectum | 15 | 1.07 | 0.97 to 1.18 | 13.00 | 0.308 |  |
| Proximal colon | 4 | 0.90 | 0.78 to 1.04 | 65.30 | 0.021 | 0.90 |
| Distal colon | 4 | 0.92 | 0.79 to 1.07 | 0.00 | 0.992 |  |
| Follow-up duration, y |  |  |  |  |  |  |
| >10 | 9 | 0.98 | 0.91 to 1.06 | 48.00 | 0.052 | 0.85 |
| ≤10 | 10 | 0.97 | 0.90 to 1.05 | 41.20 | 0.083 |  |
| Specific dietary assessment method |  |  |  |  |  |  |
| Yes | 15 | 0.99 | 0.94 to 1.06 | 45.90 | 0.027 | 0.49 |
| No | 4 | 0.91 | 0.81 to 1.02 | 0.00 | 0.412 |  |
| Subtypes of coffee |  |  |  |  |  |  |
| Caffeinated coffee | 3 | 0.99 | 0.90 to 1.10 | 54.60 | 0.086 | 0.34 |
| Decaffeinated coffee | 3 | 0.89 | 0.80 to 0.99 | 2.30 | 0.381 |  |
| Publication year |  |  |  |  |  |  |
| Before 2000 | 5 | 0.89 | 0.80 to 0.99 | 0.00 | 0.554 | 0.19 |
| 2000-2014 | 14 | 1.00 | 0.94 to 1.07 | 46.30 | 0.029 |  |
| Controlling smoking in models |  |  |  |  |  |  |
| Yes | 13 | 0.96 | 0.91 to 1.02 | 43.30 | 0.048 | 0.63 |
| No | 6 | 1.06 | 0.91 to 1.24 | 39.40 | 0.143 |  |
| Controlling alcohol in models |  |  |  |  |  |  |
| Yes | 11 | 0.95 | 0.90 to 1.01 | 45.30 | 0.050 | 0.27 |
| No | 8 | 1.07 | 0.95 to 1.21 | 27.00 | 0.213 |  |
| Controlling physical activity in models |  |  |  |  |  |  |
| Yes | 11 | 0.97 | 0.90 to 1.03 | 38.30 | 0.094 | 0.40 |
| No | 8 | 0.99 | 0.90 to 1.09 | 51.20 | 0.045 |  |
| Controlling BMI in models |  |  |  |  |  |  |
| Yes | 15 | 0.96 | 0.91 to 1.02 | 36.90 | 0.075 | 0.32 |
| No | 4 | 1.13 | 0.94 to 1.36 | 47.90 | 0.124 |  |
| Controlling energy intake in models |  |  |  |  |  |  |
| Yes | 5 | 0.97 | 0.89 to 1.05 | 53.90 | 0.070 | 0.63 |
| No | 14 | 0.98 | 0.91 to 1.05 | 40.90 | 0.055 |  |
| Controlling red, processed meat in models |  |  |  |  |  |  |
| Yes | 7 | 0.98 | 0.91 to 1.06 | 34.10 | 0.168 | 0.87 |
| No | 12 | 0.97 | 0.90 to 1.05 | 49.00 | 0.028 |  |
| Controlling dairy products, calcium intake in models |  |  |  |  |  |  |
| Yes | 5 | 0.97 | 0.89 to 1.05 | 59.30 | 0.044 | 0.54 |
| No | 14 | 0.98 | 0.91 to 1.05 | 37.60 | 0.076 |  |
| Controlling fruit, vegetables in models |  |  |  |  |  |  |
| Yes | 4 | 0.88 | 0.78 to 0.98 | 9.40 | 0.346 | 0.28 |
| No | 15 | 1.00 | 0.94 to 1.06 | 40.30 | 0.053 |  |
| Controlling folate in models |  |  |  |  |  |  |
| Yes | 2 | 1.03 | 0.84 to 1.26 | 0.00 | 0.967 | 0.69 |
| No | 17 | 0.97 | 0.92 to 1.03 | 47.30 | 0.016 |  |

*The relative risk was pooled by using fixed effects meta-analysis. Abbreviations: BMI, body mass index.
